# Non-AIDS defining cancer (NADC) among HIV-infected patients at an oncology tertiary-care center in Mexico

**DOI:** 10.1186/s12981-018-0202-2

**Published:** 2018-10-27

**Authors:** P. Cornejo-Juárez, D. Cavildo-Jerónimo, P. Volkow-Fernández

**Affiliations:** Infectious Diseases Department, Instituto Nacional de Cancerología (INCan), Av. San Fernando No. 22 Col. Sección XVI, Del. Tlalpan, 14000 Mexico City, CDMX Mexico

**Keywords:** Non-AIDS defining cancer, HIV, Mortality, HPV

## Abstract

**Background:**

Non-AIDS defining cancers (NADCs) have been an increasing cause of morbidity and mortality in patients with HIV. There is no data on the spectrum of NADCs in Mexico. We describe the type of neoplasms, clinical characteristics, and outcomes of HIV-infected patients with NADCs.

**Methods:**

We conducted a retrospective study of all patients with confirmed diagnosis of NADC attending the HIV/AIDS clinic at the National Cancer Institute in Mexico City (a tertiary-care center for adult patients with cancer) from January 1990 to December 2016.

**Results:**

From 1126 HIV-positive individuals seen at the institute since 1990, 127 (11.3%) were diagnosed with NADCs; seven patients developed two NADCs during their follow-up. At diagnosis of NADC median age was 43.7 ± 10.9 years; 101 (79.5%) were male; median CD4 was 273 cells/mm^3^, 70 patients had a CD4 count of > 200 cells/mm^3^, 73 had undetectable HIV viral load and 82 had taken combined antiretroviral therapy (cART) for more than 1 year. The most frequent NADCs were in men, Hodgkin lymphoma (34.3%) followed by anal cancer (15.7%), whereas in women, were vulvo-vaginal cancers associated to human papilloma virus (HPV) (51.8%), followed by breast cancer (25.9%). The main risk factor associated with death was cancer progression or relapse (OR, 28.2, 2.5–317.1; *p* = 0.007).

**Conclusions:**

HL- and HPV-related neoplasms are the commonest NADC in a cancer referral hospital from a middle-income country with universal access to cART since year 2005. Screening for early anogenital lesions should be emphasized in patients with HIV. It is essential to establish multidisciplinary groups involving Hemato-oncologists, Oncologists, Gynecologists, and HIV Specialists in the treatment of these patients.

## Background

Cancer is the main cause of death among human immunodeficiency virus (HIV)-infected patients with access to combined antiretroviral therapy (cART) [1]. For some neoplasms such as lung and anal cancer, these occur at an early age and may have a more aggressive presentation in HIV patients than in general population [2]. Historically, AIDS-defining cancers (ADC), especially Kaposi sarcoma (KS) and non-Hodgkin lymphoma (NHL) accounted for a substantial proportion of the morbidity and mortality in HIV-infected individuals; with the use of cART, the incidence of these malignancies has decreased [1, 3]. However, there has been an increase in Non-AIDS Defining Cancers (NADCs) from 1.6/1000 persons-years in 1999–2000 to 2.1 in 2009–2011 [4]; this higher incidence has been observed after extended use of cART [5]. Immunologic status is not closely linked with NADCs; other factors that have been implicated, such as tobacco smoking and chronic co-infections with potentially oncogenic pathogens (*Helicobacter pylori*, hepatitis B virus [HBV], hepatitis C virus [HCV], Epstein–Barr virus [EBV], and human papilloma virus [HPV]) [4].

In Mexico, 9300 new HIV cases were reported in 2015, with a mortality rate of 3.9/100,0000 persons per year [6]. Universal access to cART began in 2005, with a significant decrease in ADC, but there are, to our knowledge, no data related to the spectrum of NADCs. The purpose of this work was to describe the epidemiology, clinical characteristics, risk factors, and outcome of HIV-infected patients with NADCs at a referral center of cancer in Mexico City.

## Methods

The study was approved by the INCan Institutional Review Board (Rev/13/17). Due to the observational and retrospective nature of the study, a waiver of informed consent was granted. Authors involved in data analysis could not identify individual patients since the database used study-specific numbers. The study included all patients who had been seen at the HIV/AIDS Clinic with a diagnosis of NADC, from January 1990 to December 2016, at a referral center for adult patients with cancer in Mexico City, Mexico. Patients may previously have the diagnosis of HIV and be referred to the hospital with the NADC diagnosis, or they could be followed at our hospital because a previous diagnosis of ADC and develop the NADC during their follow-up, or were sent to our hospital with cancer and the diagnosis of HIV was made during the diagnostic approach.

We included all patients with a NADCs confirmed by histopathology during the previous 6 months of HIV diagnosis or afterwards. Patients who had benign tumors, those with ADC, or those without follow-up (less than two visits to the hospital), were excluded.

The following sociodemographic and clinical variables were recorded: age, gender, history of tobacco smoking or alcohol consumption, route of HIV infection, CD4 count and viral load at HIV diagnosis and at NADC diagnosis, co-infection with HBV or HCV, current and previous cART, tumor status at last visit (progression, relapse, stable disease, complete or partial remission) and final outcome defined as alive; death related to HIV; death related to cancer, and death from another cause. For women with HPV-related cancer, we also recorded the history of HPV cervical lesions. Pre-cART period was considered from 1996 to 2004, and cART period from 2005 to 2016.

## Definitions

- AIDS-defining cancer (ADC): malignancies that occur in HIV-infected persons categorized as an AIDS-defining event (KS, NHL, cervical cancer).
- NADC: cancer types not included as part of ADC. NADC mainly include Hodgkin lymphoma (HL), cancers of the mouth, throat, liver, lung, vulvo-vagina, and anus, among others [7].
- Tobacco smoker: has smoked at least 100 cigarettes during his lifetime, smokes daily or is current smoker.
- Moderate or heavy alcohol consumption: Men consuming more than two alcohol-containing beverages per day, or women consuming more than one alcohol-containing beverage per day [8].
- HBV co-infection: (a) replicative HBV: HBsAg or HBeAg-positive; (b) non-replicative HBV: HBsAg, and HBc AB-positive.
- HCV co-infection: at least two positive HCV serologies or an HVC viral load with > 20 copies/mL.
- Cancer status: (a) complete remission: Disappearance of all the signs and symptoms of cancer; (b) partial remission: some, but not all, signs and symptoms of cancer have disappeared; (c) stable disease: cancer that is neither decreasing nor increasing in extent or severity; (d) relapse: return of the disease or of the signs and symptoms of a disease after a period of improvement; (g) progression: disease becomes worse or spreads in the body [9].
- cART: combination antiretroviral therapy that suppresses HIV replication.
- WHO/REAL (World Health Organization Classification/Revised European American Lymphoma) HL classification: (A) nodular lymphocyte predominance, and (B) classical HL: (a) nodular sclerosis HL; (b) lymphocyte-rich classical HL; (c) mixed cellularity HL, and (d) lymphocyte-depletion HL.
- Death related to HIV: death related with an opportunistic disease or related with ADC in a patient without cART or in a patient receiving cART in whom viral suppression and immunologic recovery has not been achieved.
- Death related with cancer: death due to progression or relapse of neoplastic disease.
- Death related to another cause: death related to a non-HIV and non-cancer disease.

## Statistical analysis

Quantitative variables were calculated as mean ± standard deviation (SD), or median and InterQuartile Range (IQR) as appropriate. Logistic regression analysis for mortality included the following variables considered as potential risk factors: age (≥ 50 years); history of smoking; having received more than one cART regime; history of opportunistic infections; HBV or HCV co-infection; cancer stage III or IV; tumor progression or relapse, and hematologic cancer and CD4 count ≤ 200 cells/µL at cancer diagnosis and during the pre- or post-cART period. Log-rank test and Kaplan–Meier were performed for mortality. Data was analyzed using STATA ver. 14 (Stata Corp., College Station, TX, USA) statistical software.

## Results

During the study period, 1126 patients were seen at the HIV/AIDS Clinic; 142 had a diagnosed NADC (12.6%). Ten patients were excluded: eight with cancer diagnosed 6 months before HIV, and two with fewer than two hospital visits. There were 134 NADC that presented in 127 patients (seven had two different neoplasms).

Median age at cancer diagnosis was 43.7 ± 10.9 years, 101 (79.5%) were men. Sixty-seven patients (52.7%) had a history of smoking and 67 (52.7%) had a history of heavy alcohol consumption. Five patients (3.9%) had replicative HBV, 21 (16.5%) had non-replicative HBV, and eight (6.1%) had HCV co-infection.

Of the whole group, 26 patients (20.5%) were diagnosed prior to the cART era: 12 (46.2%) had cancer relapse or progression: three hematologic malignancies (60%) and nine solid tumors (42.9%). Fourteen (53.8%) had stable disease or complete remission: two hematologic malignancies (40%) and 12 (57.1%) with solid tumor (p = 0.634).

**Fig. 1:**
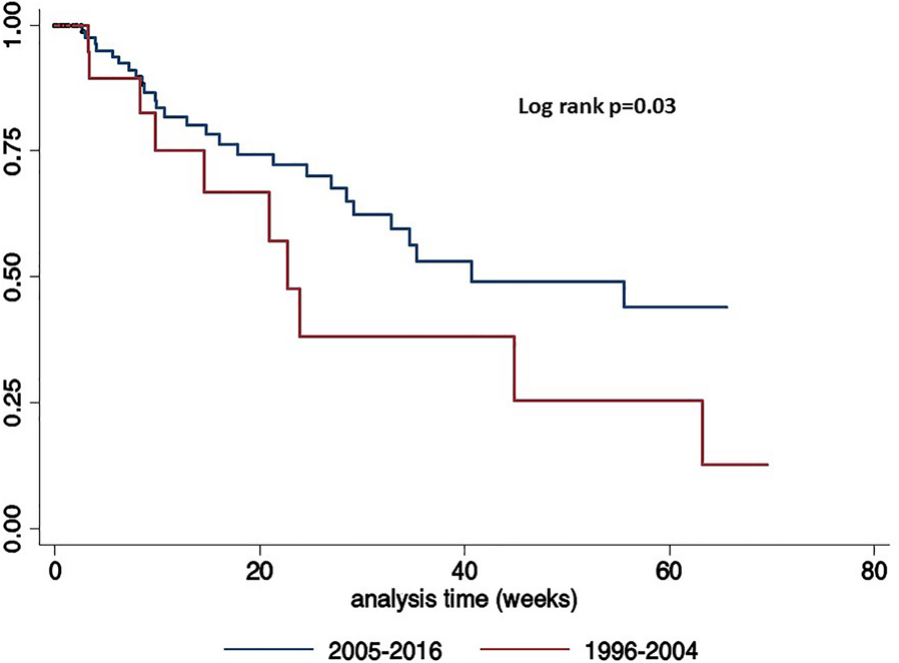
Kaplan–Meier curve showing patients with relapse or progression since NADC diagnosis until last hospital visit, according to pre-cart (1996–2004) and post-cART (2005–2016)

One hundred six patients (80.5%) were diagnosed during the cART era: 26 (24.5%) had progression or relapse of neoplastic disease: four with hematologic malignancies (10.8%) and 22 with solid tumor (31.9%). Eighty (75.5%) had stable disease or were in clinical remission (n = 33, 89.2% with hematologic malignancies and n = 47, 68.1% with solid tumor, p = 0.01). There was a significant increase risk in relapse or progression in pre-ART era when compared to c-ART period (OR, 2.63; 95% CI 0.97–7.0; *p* = 0.03), as it is shown in Fig. 1.

The median of CD4 at HIV diagnosis was 132 cells/µL (IQR 47; 278) and the median of viral load 98,922 copies/mL (IQR 37,053; 413,856). At cancer diagnosis, 70 patients had a CD4 count of > 200 cells/µL with a median of 273 cells/µL (IQR 125; 481), and 73 patients had undetectable HIV viral load. In those patients who were not undetectable (*n* = 43) the median viral load was 37,053 copies/mL (IQR 4950; 107,064) (Table 1).

**Table 1:** Clinical characteristics of 127 HIV-infected patients with non-AIDS defining cancer (NADC)

Characteristics—N (%)	Total	Women (*n* = 26, 20.5)	Men (*n* = 101, 79.5)
Age (years)^a^	43.7 ± 10.9	49 ± 11.1	42.3 ± 10.5
BMI	23.9 ± 4	24.6 ± 3.9	23.7 ± 4
Smoking	67 (52.7)	9 (34.6)	58 (57.4)
Alcohol consumption	68 (53.5)	6 (23)	62 (61.4)
HIV transmission mode			
MSM	65 (51.2)	0	65 (64.3)
Heterosexual	55 (43.3)	22 (84.6)	34 (33.7)
Transfusion	2 (1.6)	1 (3.8)	1 (1)
Unknown	4 (3.1)	3 (11.5)	1 (1)
cART at NADC diagnosis	93 (73.2)	21 (80.7)	74 (73.3)
NRTI + NNRTI	54 (58)	6 (2.9)	48 (64.9)
NRTI plus PI/r	29 (31.2)	9 (4.3)	20 (27)
NRTI + NNRTI + PI/r	5 (5.4)	3 (1.4)	2 (2.7)
Other	7 (7.5)	3 (1.4)	4 (5.4)
CD4 (cells/µL) at nadir^bc^	132 (47, 278)	106 (12, 249)	145 (50, 284)
< 200	40 (61.9)	6 (66.7)	34 (61.8)
> 200	24 (38.1)	3 (33.3)	21 (38.2)
Viral load at HIV diagnosis (copies/mL)^cd^	98,922 (37,053; 413,856)	307,395 (26,676; 875,000)	98,922 (37,495; 242,356)
CD4 at NADC diagnosis^de^	273 (125; 481)	477 (300; 805)	231 (114; 380)
< 200	44 (38.5)	5 (20)	39 (43.8)
> 200	70 (61.4)	20 (80)	50 (57.3)
Undetectable viral load at NADC diagnosis^f^	73 (65.7)	21 (84)	52 (60.4)
Viral load at NADC diagnosis^cg^	37,053 (4950; 107,064)	30,915 (145; 37,053)	39,951 (8751; 109,072)

Percentages for each combination were obtained with the 93 patients who were receiving cART

BMI, body mass index; MSM, men who have sex with men; cART, complete antiretroviral treatment; NRTI, nucleoside reverse transcriptase inhibitors; NNRTI, non-nucleoside reverse transcriptase inhibitors; PI/r, protease inhibitors plus ritonavir

^a^Mean ± standard deviation (SD)

^b^CD4 count was available in 64 patients

^c^Median (IQR range)

^d^Viral load was performed in 53 patients at HIV diagnosis (eight women and 45 men)

^e^114 patients had CD4 count at NADC diagnosis

^f^Viral load was performed in 116 patients at NADC diagnosis (28 women and 88 men)

^g^Median viral load was calculated in those patients who were not undetectable (n = 43, 7 women and 36 men)

**Fig. 2:**
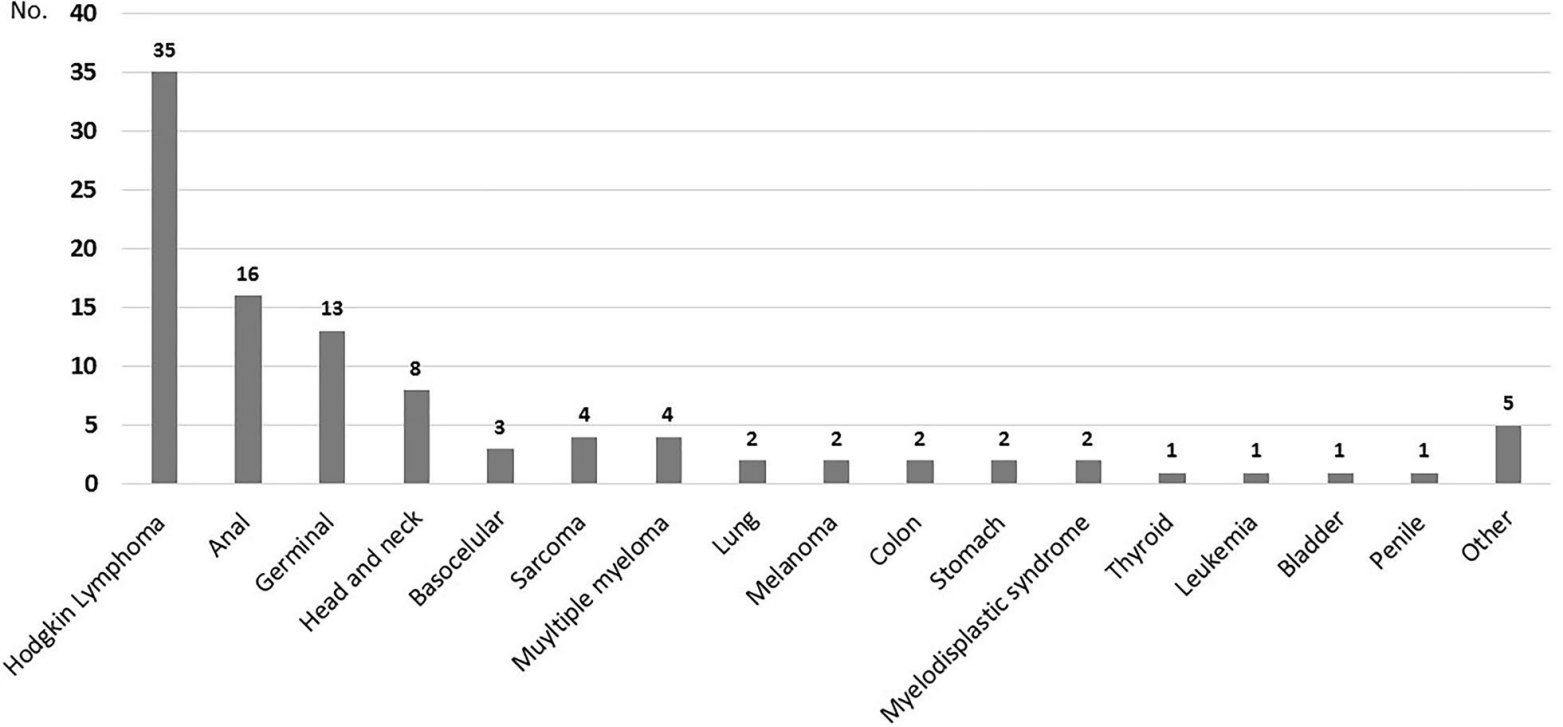
Distribution of Non-AIDS Defining Cancer (NADC) in men (*n* = 101). One patient had two different neoplasms. Other cancers included one chondrosarcoma, one ampulla of Vater, three epidermoid, and one giant cell tumor

In men (*n* = 101), the most frequent NADC was HL (*n* = 35, 34.6%), followed by anal cancer (*n* = 16, 15.8%), and germinal tumors (*n* = 13, 12.9%). Two patients (2%) had melanoma, two (2%) had lung cancer, and three (2.4%) had skin basocellular cancer. One patient had two neoplasms during his follow-up: HL and lung cancer. The remainder type of the tumors in men are presented in Fig. 2.

**Fig. 3:**
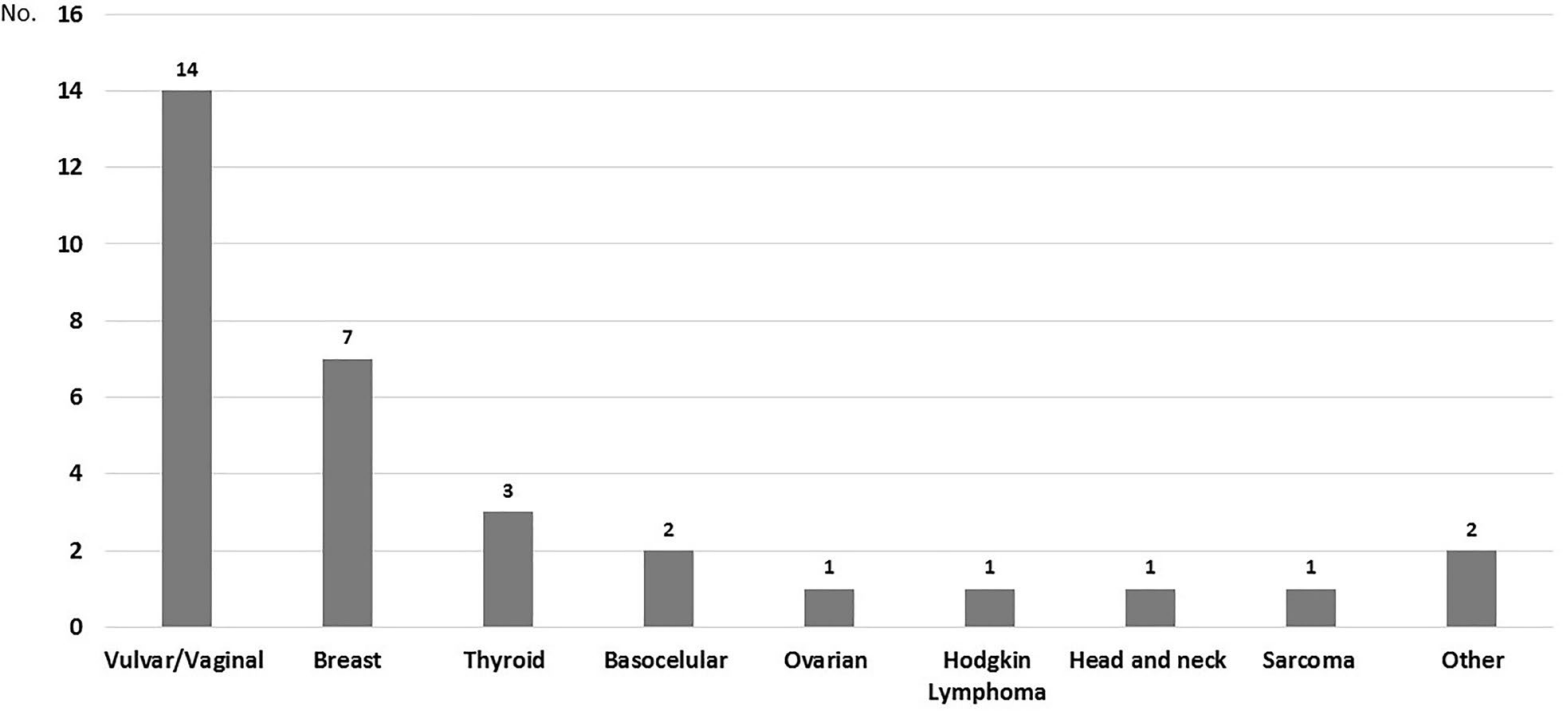
Distribution of Non-AIDS Defining Cancer (NADC) in women (*n* = 26). Four patients had two different neoplasms. Other cancers include one unknown and one epidermoid

Twenty-six were in women (20.5%), of them 13 had vulvo-vaginal cancer associated to HPV (50%), seven had breast cancer (26.9%), and two had basocellular skin cancer (7.7%). Six patients had two different neoplasms: one vaginal and thyroid, one chondrosarcoma and epidermoid, one vulvar and vaginal, one vulvar and ovarian, one vulvar and basocellular, and one, vulvar and breast cancer (this latter patient is alive and with no tumor activity, both cancers had been diagnosed 14 and 15 years, respectively, after she was treated for a NHL). Figure 3 presents all of the cancers in the women’s group.

Of the 13 patients with vulvar and/or vaginal cancers, in seven, the diagnosis was performed during the annual colposcopy screening, a program performed for HIV-positive women at the Dysplasia Clinic. One of these patients, a smoker, had cervical conization related to Low-Grade Squamous Intraepithelial Lesion (LGSIL); she had multifocal asynchronous lesions, and multiple condylomas located in cervix, vagina, vulva, and perianus, although her CD4 count has remained over 1600 CD4 cells/µL with undetectable viral load, she is an active smoker. Other characteristics in patients with vulvo-vaginal neoplasms are illustrated in Table 2.

**Table 2:** Main characteristics of 13 women with vulvar and/or vaginal HPV-related neoplasms

Number of patients	Age	CD4 at cancer diagnosis (cells/µL)	Smoking status	Previous diagnosis of dysplasia or cervical cancer^a^	Multifocal or recurrent cancer
1	35	805	–	In situ	+
2	43	1602	+	LGSIL	+
3	44	80	–	LGSIL	–
4	45	477	+	LGSIL	–
5	47	719	–	No	–
6^b^	49	645	–	No	+
7^c^	52	371	+	In situ	+
8	56	481	–	In situ	–
9	57	556	–	Clinical stage IIB	–
10	75	356	–	No	–
11	37	249	+	No	–
12	57	191	+	No	–
13	72	91	–	No	–

Diagnosis was performed by cytopathic alterations associated to HPV

^a^LGSIL: low-grade squamous intraepithelial lesions

^b^This patient had two HPV-cancers: vulvar and vaginal. She had multifocal asynchronous lesions, although her CD4 count has reached 1600 cells/µL with undetectable viral load

^c^This patient had three different neoplasms: Non-Hodgkin lymphoma, breast cancer, and vulvar cancer

HL was the most frequent malignancy in men (*n* = 35, 34.6%), 20 with mixed cellularity (57.1%); six with nodular sclerosis (17.1%); one with lymphocyte-rich (2.9%); one with lymphocyte-depleted (2.9%), and seven (20%) were classified as classic without the specific type. In 26 patients, HL was diagnosed after a median of 4.5 years (IQR 3.4; 9.4 years) of HIV diagnosis, with a mean CD4 count of 240 ± 171 cells/µL. In nine patients (26%), both diagnoses were made at the same time, with a mean of CD4 count of 162 ± 88 cells/µL.

Other hematological malignances diagnosed were multiple myeloma in four (3.1%), myelodysplastic syndrome in two (2%), and acute leukemia in one patient (1%).

Median CD4 count was the lowest in germinal tumors (117 cells/µL) and the highest in women with breast cancer (890 cells/µL); median viral load was < 40 copies/mL in nearly all patients with neoplasms, except for germinal and head-and neck-tumors. Other clinical characteristics among the main NADC reported are shown in Table 3.

**Table 3:** Clinical characteristics of the main non-AIDS defining cancer (NADC) identified

NADC —*N* (%)	HL—36 (28.3)	Anal—16 (12.6)	Germinal—13 (10.2)	Head/neck—9 (7.1)	Lung—2 (1.6)	Skin—7 (5.5)	Breast—7 (5.5)	Vulvar/vaginal—13 (10.2)^a^
Age (years)^b^	39.9 ± 9.1	42.2 ± 7.8	33.4 ± 5.5	49.1 ± 8.1	47 and 51	57.3 ± 11.3	43.2 ± 1.9	50.3 ± 10.8
CD4 (cells/µL)^c^	162 (106, 302)	270 (152, 457)	117 (33, 421)	214 (103, 513)	219 and 231	369 (249, 593)	890 (472, 927)	481 (371, 805)
Viral load (copies/mL)^c^	< 40 (< 40, 2168)	< 40 (< 40, 78,000)	51,930 (51, 750,000)	17,970 (2475, 34,205)	< 40 and 21,300	< 40 (< 40, 16,300)	< 40 (< 40, 50)	< 40 (< 40, 55)
Follow-up (weeks)^cd^	8.9 (2.7, 31)	13.9 (5.8, 30.2)	13.6 (4.3, 29.1)	10.7 (8.5, 22.8)	1.7 and 5.9	22.1 (6.5, 49.6)	9.7 (7.4, 26.8)	9.2 (3.2, 26.9)
Alive	30 (83.3)	9 (56.2)	0	8 (88.9)	1 (50)	6 (85.7)	6 (85.7)	11 (84.6)
Dead	6 (16.7)	7 (43.8)	13 (100)	1 (50)	1 (50)	1 (43.3)	1 (43.3)	2 (15.4)

HL, Hodgkin lymphoma

^a^There were 13 patients with 14 vulvar/vaginal cancers (one patient had both)

^b^Mean ± standard deviation (SD)

^c^Median (InterQuartile Range [IQR])

^d^Follow-up was from NADC diagnosis until last hospital visit

Median follow-up since HIV infection until last hospital visit was 7.3 years (IQR 2.7; 13.3); median follow-up after NADC diagnosis was 1.4 years (IQR 0.4; 3.9). In 82 patients (64.5%) NADCs were diagnosed after 1 year of cART, with a median time of 8.7 years (IQR 4, 13).

At the last visit, 51 of the patients (40.2%) had complete remission, 36 (28.3%) had stable disease, and 40 (31.5%) progression or relapse. At the time of the last hospital visit, 24 patients (19%) had died: one (0.8%) secondary to HIV infection, 14 (11%) cancer-related, and in nine patients (7.1%) deaths were associated with other causes. The main risk factor associated to mortality was progression or relapse of the malignant disease (OR 28.2, 2.5–317.1; *p* = 0.007).

## Discussion

From 1126 patients seen in the HIV/AIDS clinic of a cancer referral center in a middle-income country, 12.6% had a NADC, 80.5% were diagnosed during the cART era. The most common tumors were HL and HPV associated cancers.

In Mexico in 2016, it was estimated that 220,000 persons were HIV-infected, 12,000 new cases were diagnosed, and there were 4900 AIDS-related deaths [10]. HIV-infected patients are at significant risk for many types of cancers, such as HL, anal cancer, germinal tumors, vulvar and vaginal, basocellular skin, and breast cancer [11].

In the cART era, international epidemiological studies revealed that rates of ADC had decreased (NHL and KS declined threefold each); but this was not so for cervical cancer, which has steadily increased [5]. However, it has been reported in some studies that NADCs have increased by over threefold during the same time period [12]. In a prospective cohort study made in the US during 1997 to 2012, the authors found that all crude cancer incidence rates increased between 1997 to 2000 and from 2009 to 2012, but the age-standardized rates decreased significantly for all cancers, ADC and non-virus-related NADCs, with a borderline decrease for the rest of NADC [13]. It has been speculated that as people with HIV live longer with chronic immune suppression, the actual duration of HIV may be an important etiologic factor in the development of NADCs [14, 15].

Some studies in the US describe HL, lung, anal, and liver cancer as the most prevalent NADCs among HIV-infected patients; while other studies report breast, colon and esophageal as the most prevalent [1]. In HIV patients from the Caribbean, Central and South American network for HIV Research (CCASAnet) in 2011, ADC were the most frequent (82%), occupying KS and NHL the first two places, and from NADCs, HL and skin cancers were the most frequent, and were more likely diagnosed in older subjects. A great proportion (74%) were diagnosed more than 1 year after HIV diagnosis (CCASAnet) [15]. In 64.5% of our patients, NADC was diagnosed after at least 1 year of cART, with a median of 8.7 years. A Brazilian study reported the five most incident cancers among HIV men were KS, NHL, anal, colorectal and lung cancer but found that NADCs were most common than ADC. The prevalence of NADCs contrast to what is seen among general population in whom the five most frequent incident cancers sites were prostate, colon-rectum, lung, stomach and skin non-melanoma [16].

In women with HIV, cervical cancer, NHL and breast cancer were the most incident cancers. In contrast, among women from the general population, breast, colon-rectum, thyroid, cervix and skin non-melanoma were the most frequent sites [16].

In the present study, the prevalence and outcome of NADCs, at an oncological referral center in a middle-income country, were similar as reported in other countries, being HL the most frequent NADC in males; although in females, vulvar and vaginal were the most common (53.8%). Considering HL (EBV-related), and anal, vulvar and vaginal cancer (HPV-related), virus-associated tumors represented 50% of all neoplasms in this study.

In several reports, NADCs are diagnosed at a younger age in HIV-infected patients when compared with general population; the risk of developing a NADC increases with age, being 12 times more frequent in HIV-infected patients older than 40 years [12]. Mean age in our whole cohort was 43 years but, on analyzing each NADC separately, patients with germinal tumors were the youngest (33 years) and skin-cancer patients the oldest (57 years).

In this cohort, we found a significant increase in CD4 count at the time of NADC diagnosis (273 cells/µL) as compared to CD4 at nadir (132 cells/µL), usually when HIV is diagnosed, suggesting that low CD4 does not predict the occurrence of malignancies.

HL was the most frequent NADC (*n* = 36, 28.3%). Some epidemiological studies showed that patients with HIV have a tenfold risk of HL compared with HIV-negative subjects; incidence increased after the introduction of cART. HL displays an unusually aggressive behavior, advanced stages, extranodal involvement, more aggressive subtypes, and an overall poor prognosis [11]. In patients with HL in whom HIV and HL were diagnosed simultaneously, the CD4 count was lower than in patients in whom the HL diagnosis was done in patients already on cART (162 ± 88 cells/µL vs. 240 ± 171 cells/µL). In a study of 848 patients with HIV-HL, 30% were mixed cellularity, 30% nodular sclerosis, and 38% classical HL not otherwise specified [11, 17]. The main histologies that we found were mixed cellularity (58%), nodular sclerosis (16%), and classical without a subtype specified (19%). Ninety-four percent of our patients were classified in stages III or IV, all of had B symptoms, and six (16.7%) patients died.

HPV prevalence in Mexican women with HIV has been reported to be over 70% [18], HPV-type-associated-cancers entertain an increased risk of in situ and invasive cancer, with no association to the degree of immunosuppression [4, 18, 19]. Prevalence of cervical HPV infection in women with normal cervical cytology among HIV-infected women in Latin America, has been estimated to be 57%, but in Brazil was as high as 84% [20]. Ten patients with vulvar and/or vaginal cancer were diagnosed during the annual colposcopic screening, and four were referred because of vulvar or vaginal neoplasms. All HIV-infected women seen in our Dysplasia Clinic are followed with annual colposcopy screening. The persistence of HPV lesions could be associated with immunosuppression, but other risk factors exist, such as cigarette smoking [19]. One of the patients described in this series had multiple recurrences of vulvar multifocal cancer, having as main risk factor smoking; she had over 1600 CD4 cells/µL when first diagnosed, was on cART with undetectable viral load and high CD4 counts for over 15 years.

Anal cancer has an aggressive clinical outcome and a poor prognosis [21]. In this cohort, 16 men (all Men who have Sex with Men—MSM) had anal cancer, significantly younger (mean age, 42 years) compared with general population (60 years) [11, 17]. Seven of these patients died (44%), highlighting the need for screening procedures for HPV related lesions in all HIV-infected patients for early detection of HPV-related anal/genital lesions [22].

Lung cancer has also been reported in HIV-infected patients; the risk has been estimated as more than two-to-sixfold higher compared with general population, closely related to high rates of smoking in HIV-infected patients (40–70%) compared with non-HIV population (20%) [4, 17]. Non Small-Cell Lung Cancer (NSCLC) is the main histologic type, and it has short survival (1–4 months) in advanced stage. Severe immunodeficiency could be a significant risk factor, as CD4 count at the time of NSCLC diagnosis had been reported between 120 and 288 cells/µL. We report only two cases of NSCLC with CD4 count of 219 and 231 cells/µL, and viral load of < 40 and 21,300 copies/mL, respectively, at cancer diagnosis (both patients smoked tobacco).

We documented five cases of basocellular cancer and two cases of melanoma, representing 5.5% of the total NADCs [3, 23, 24].

Prevalence of co-infection with HBV was 3.9, and 6.3% for HCV, significantly lower than in Southeast Asia, sub-Saharan and central Africa areas that had reported a prevalence as high as 30% [17, 25] and similar to the 3.3% for HBV and 6% for HCV in Mexican general population [26].

We did not observe HIV-infected subjects with hepatocellular carcinoma despite threefold to sixfold reported excess risk when compared with general population [4].

Breast cancer became the first cause of cancer-related deaths in Mexican women [24]. We report seven women with breast cancer, with a median CD4 count of 481 cells/µL and undetectable median viral load. To our knowledge, until date, there is no relationship between HIV infection and its occurrence of breast cancer.

Testis cancer, in particular seminoma and extragonadal germ cell cancer occur with a slightly greater risk in HIV-infected patients, without relation to CD4 count (two large series with 34 and 35 patients each one, reported the median CD4 count at cancer diagnosis was 325 and 315 cells/µL, respectively) [27]. Germ-cell tumors ranked third in frequency in males in our series (10.7%); mean age was 33 years, similar to the age reported in general population (25–45 years). Patients were diagnosed with lower CD4 count (median 117 cells/µL) and high viral load (median 51,930 copies/mL).

Primary prevention measures are crucial in order to obtain a decrease in cancer incidence. Universal HBV vaccination has impacted in hepatocellular carcinoma in countries that have implemented this policy for a long time [28]. Primary prevention of cervical cancer including HPV vaccination and screening programs that include Pap-smear cytology and/or HPV test as secondary prevention [28], have allowed the increase of the timely detection of neoplasms associated with this virus. HPV anal cytology has also demonstrated a positive impact on reducing anal cancer in MSM [22]. A cornerstone preventive strategy is smoking cessation, and others include avoiding ultraviolet (UV) exposure, moderate alcohol consumption, and eradication of *Helicobacter pylori* infection [28].

This study has some limitations, because we included only patients from a single cancer referral center in Mexico City, receiving patients from central part of the country. Being a retrospective study, there can be selection and information biases. On the other hand, we report a well-defined group of patients with prolonged follow-up and, from our perspective, describing adequately the spectrum of cancer in HIV patients from a middle income country with cART.

## Conclusions

HL- and HPV-related neoplasms are the commonest NADC in a cancer referral hospital from a middle-income country with universal access to cART since year 2005. Screening for early anogenital lesions should be emphasized in patients with HIV. It is essential to establish multidisciplinary groups involving Hemato-oncologists, Oncologists, Gynecologists, and HIV Specialists in the treatment of these patients.

## Abbreviations

cART: combined antiretroviral therapy
EBV: Epstein–Barr virus
HBV: hepatitis B virus
HCV: hepatitis C virus
HIV: human immunodeficiency virus
HL: Hodgkin lymphoma
HPV: human papilloma virus
KS: Kaposi’s sarcoma
MSM: men who have sex with men
NHL: non-Hodgkin lymphoma
WHO/REAL: World Health Association Classification/Revised European American Lymphoma
